# How Illusory Is the Solitaire Illusion? Assessing the Degree of Misperception of Numerosity in Adult Humans

**DOI:** 10.3389/fpsyg.2016.01663

**Published:** 2016-10-27

**Authors:** Christian Agrillo, Audrey E. Parrish, Michael J. Beran

**Affiliations:** ^1^Department of General Psychology, University of PadovaPadova, Italy; ^2^Language Research Center, Georgia State UniversityAtlanta, GA, USA

**Keywords:** numerosity illusion, approximate number system, visual illusions, quantity estimation

## Abstract

The Solitaire illusion occurs when the spatial arrangement of items influences the subjective estimation of their quantity. Unlike other illusory phenomena frequently reported in humans and often also in non-human animals, evidence of the Solitaire illusion in species other than humans remains weak. However, before concluding that this perceptual bias affects quantity judgments differently in human and non-human animals, further investigations on the strength of the Solitaire illusion is required. To date, no study has assessed the exact misperception of numerosity generated by the Solitaire arrangement, and the possibility exists that the numerical effects generated by the illusion are too subtle to be detected by non-human animals. The present study investigated the strength of this illusion in adult humans. In a relative numerosity task, participants were required to select which array contained more blue items in the presence of two arrays made of identical blue and yellow items. Participants perceived the Solitaire illusion as predicted, overestimating the Solitaire array with centrally clustered blue items as more numerous than the Solitaire array with blue items on the perimeter. Their performance in the presence of the Solitaire array was similar to that observed in control trials with numerical ratios larger than 0.67, suggesting that the illusory array produces a substantial overestimation of the number of blue items in one array relative to the other. This aspect was more directly investigated in a numerosity identification task in which participants were required to estimate the number of blue items when single arrays were presented one at a time. In the presence of the Solitaire array, participants slightly overestimated the number of items when they were centrally located while they underestimated the number of items when those items were located on the perimeter. Items located on the perimeter were perceived to be 76% as numerous as centrally located items. The magnitude of misperception of numerosity reported here may represent a useful tool to help to understand whether non-human animals have different perceptual mechanisms or, instead, do not display adequate numerical abilities to spot the illusory difference generated in the Solitaire array.

## Introduction

Visual illusions are commonly used in perception studies as they can reveal how visual information is processed in terms of top-down and bottom-up mechanisms. These illusions also are useful because such information cannot be obtained by the exclusive study of photoreceptors and neural pathways of vision (Eagleman, 2001). Interestingly, not all people perceive visual illusions in the same way. Polymorphic responding in the presence of illusory patterns, such as the Rotating-Snakes illusion and the Rotating-Tilted-Lines illusion, has been described (e.g., Billino et al., 2009). Fraser and Wilcox (1979) reported that not all human participants perceived illusory movement and also found a significant parent-offspring correlation in the response to a motion illusion, which points to the existence of either a genetic basis or a common environment effect in the emergence of visual illusions.

Comparative researchers have focused their attention on how non-human animals perceive illusory patterns. Indeed, like human perception, the perception of non-human species is likely to be subjective, in which a subject’s perception of the external world is not congruent with reality. The comparative study of visual illusions also has become a useful tool to investigate whether perceptual systems are similar/dissimilar to those described in humans. Comparative psychologists have reported that several non-human animals are sensitive to static illusions that produce misperception of size, depth or brightness: chimpanzees and monkeys, for instance, perceive the Delboeuf illusion (Parrish and Beran, 2014; Parrish et al., 2015), chicks perceive the Ebbinghaus illusion (Rosa Salva et al., 2013), baboons perceive the corridor illusion (Barbet and Fagot, 2002) and Zöllner illusion (Benhar and Samuel, 1982), while guppies are sensitive to the brightness illusion (Agrillo et al., 2016). Perception of motion illusions have been reported too, with rhesus monkeys (Agrillo et al., 2015a) and guppies and zebrafish (Gori et al., 2014) showing a human-like perception of the Rotating Snake illusion. These studies suggest perceptual systems may be more shared among vertebrates than previously thought (e.g., Nieder, 2002; Kelley and Kelley, 2014), even though no firm conclusion can be taken at this stage as several illusory phenomena have not been studied in species other than humans, and other illusory stimuli produce different outcomes across species in terms of who sees those illusions (for a review, see Feng et al., 2016).

**FIGURE 1:**
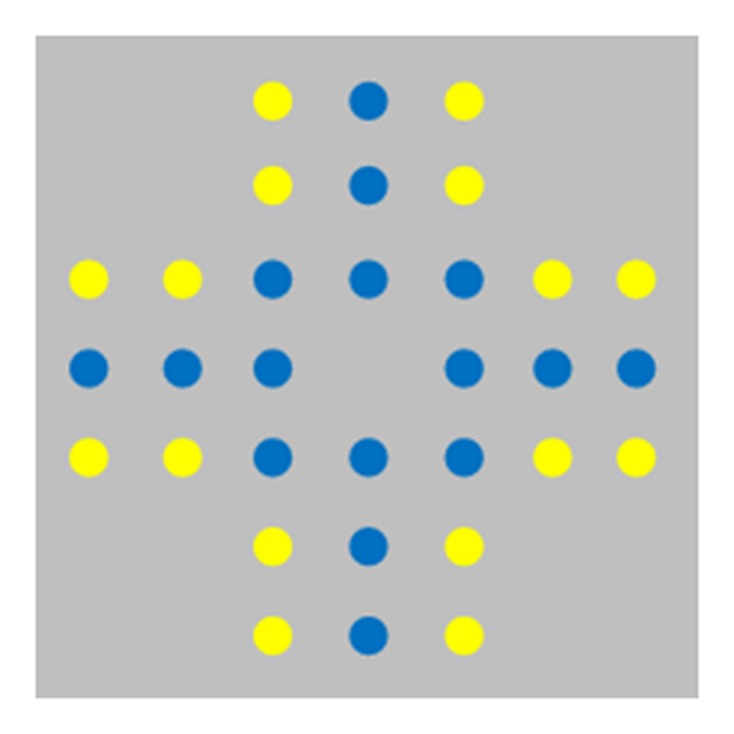
**The Solitaire illusion.** Although the same number of yellow and blue items is presented in the array, it appears for many humans that there are more blue items than yellow items.

One category of illusory patterns consists of “numerosity illusions.” In these cases, misperceptions of numerosity occur through underestimation or overestimation, depending on the spatial arrangement of the items presented in the visual scene. For instance, in the regular random numerosity illusion humans typically overestimate the number of items presented in regular patterns compared with the same number of items that are randomly distributed (Ginsburg, 1980). The Solitaire illusion, initially studied by Frith and Frith (1972), is a similar numerosity illusion and occurs when one misperceives the relative number of two different colors of otherwise identical items in intermingled sets. In the original investigation of this illusion, seven spatial arrangements were presented, 6 of them made by 24 dots (12 white and 12 black) and one made by 32 dots (16 white and 16 black). This latter arrangement is shown in **Figure 1**. For most people, the blue dots in the figure appear to be more numerous even though the array includes the same number of yellow and blue dots. This illusion seems to be primarily determined by the Gestalt law of ‘proximity’ (items that are close together tend to be grouped as part of the same object) and ‘good continuation’ (items that are arranged in a straight line tend to be grouped; Wertheimer, 1938). Because of these principles, centrally located items would form a single unit, a Gestalt, while items located in the perimeter would form four small clusters. Items forming a better Gestalt tend to be overestimated (Frith and Frith, 1972), although the exact mechanisms underlying this perceptual bias in this numerosity judgment is unknown. One potential explanation may involve the occupancy model of numerical processes (Allik and Tuulmets, 1991). According to this model, each item to be enumerated is supposed to have an impact upon its spatial neighborhood in a constant occupancy radius; the array with the larger occupancy value is perceived to be more numerous. If two items are close enough to each other (like the 16 blue items forming a single Gestalt in the Solitaire array) the territories they occupy overlap, thus leading to an overestimation of numerosity compared to when items are partially separated and their territories do not always overlap (like the case of the yellow items spatially isolated in four clusters in **Figure 1**).

Testing the perception of numerosity illusions in non-human animals could be very useful to understand the similarities and differences in perceptual factors underlying quantity estimation across vertebrates. Indeed, while it is widely recognized that vertebrates share an approximate number system (e.g., Nieder and Miller, 2004; Abramson et al., 2011; Beran and Parrish, 2016) that enables them to discriminate between differently sized groups of biologically relevant stimuli (e.g., pieces of food, number of predators, etc.), the perceptual mechanisms underlying quantity estimation are still largely unknown. Recently, the Solitaire illusion has been investigated in non-human animals, giving rise to intriguing species-level and individual-level differences. Two different experimental paradigms (spontaneous manual choice tests and computerized quantity discrimination procedures) were used to assess this illusion in non-human primates and human children and adults (Agrillo et al., 2014a; Parrish et al., 2016). In one test with chimpanzees (Agrillo et al., 2014a), two arrays made of intermixed preferred food items (blue M&Ms) and non-preferred food items (yellow cereal pieces) were presented. When real differences in the number of items were present in the arrays, the chimpanzees chose the array with more of the preferred food type. When both arrays were presented in the Solitaire arrangement, one array contained preferred food items that were centrally located and non-preferred food items that were presented on the perimeter. In the other array, the position of preferred and non-preferred food items was reversed. The assumption was that, if the chimpanzees were susceptible to the Solitaire illusion, they should have spontaneously selected the array in which preferred food items were centrally located as those items should have appeared to be more numerous (Agrillo et al., 2014a). Chimpanzees did not show any evidence for perception of this illusion.

Agrillo et al. (2014a) presented dot arrays in a computerized format to rhesus monkeys. The monkeys did not show a consistent pattern of choosing one Solitaire arrangement over the other, whereas adult humans selected the array with the centrally located arrangement of the focal dot color as predicted. A similar result was observed with capuchin monkeys, in which overall perception of the Solitaire illusion emerged at the group level but there was only weak evidence of the Solitaire illusion at the individual level (Parrish et al., 2016). Furthermore, preschool children displayed an age effect for perception of the Solitaire illusion in which older children perceived the illusion more readily than younger children (Parrish et al., 2016). This result may imply that the susceptibility to grouping cues underlying the Solitaire illusion increases throughout development, even though the possibility exists that older children were more sensitive to this illusion because their numerical acuity was more mature compared to younger children (e.g., Halberda and Feigenson, 2008). A very recent study using a two-choice discrimination task reported a similar result to that from monkeys in a more-distantly related species to humans, the guppy, in which only some fish perceived the Solitaire illusion but other individuals did not (Miletto Petrazzini et al., under review).

These differences in perception of the Solitaire illusion for humans and non-human animals are intriguing given that these same species seem to perceive other visual illusions commonly studied in humans. One might be tempted to conclude that perceptual biases affecting quantity judgments with the Solitaire arrangement in humans and other species are unrelated. However, no study has assessed the illusory numerical ratio between the number of items located in the center arrangement vs. the number of items located in the perimeter arrangement (i.e., the magnitude of the illusion in terms of dot quantity estimation). Thus, the possibility remains that the subjective difference generated by the illusory pattern is too subtle to be detected by non-human animals because they lack the perceptual sensitivity to detect small numerical differences. For instance, if the illusory ratio between items located on the perimeter and those centrally located was equal to 0.88 (i.e., being presented with 16 central items and 16 perimeter items led to misperceptions of “seeing” 17 and 15 items), animals that are known to have a lower numerical acuity (such as salamanders that typically discriminate between two quantities up to 0.67 ratio, Uller et al., 2003; Krusche et al., 2010) could not be investigated in this research field. This lack of information prevents us from drawing any conclusion about similarities/differences in the perceptual bias affecting quantity judgments for Solitaire stimuli in human and non-human animals.

To address this issue, we designed two tasks to assess the subjective ratio that is experienced by humans when seeing centrally located items and those presented in the perimeter of the Solitaire array (32 item version). The first goal of the relative numerosity task was to establish whether adult human participants perceived the Solitaire illusion with the experimental procedure and stimuli used in comparative research. To achieve this goal, participants were presented with two arrays comprised of blue and yellow items and were required to select the one that they perceived to contain the larger number of blue items. In addition, we estimated the illusory ratio for these participants between the number of dots located in the center vs. the number of items located on the perimeter. We did this by comparing performance in the presence of true numerical differences (control trials) and test trials using equal sets (Solitaire arrays). The main purpose of the numerosity identification task was to better understand the relative difference in the number of items perceived in the Solitaire illusion in each of its arrangements (with focal colored items located centrally or located on the perimeter). Unlike the relative numerosity task, here participants saw one array at a time and were required to report the number of items of a given color that they judged to be present in each array. This design aimed at assessing the exact quantities that participants believed they had seen for the various stimulus arrangements.

We believe that understanding these subjective quantitative ratios for objectively identical numbers of items will help us to better understand the reasons underlying the differential performance reported by non-human animals and humans with the Solitaire illusion (Agrillo et al., 2014a; Parrish et al., 2016; Miletto Petrazzini et al., under review). More generally, these data may provide a threshold of numerical acuity that subjects must be capable of experiencing in order to be susceptible to this numerosity illusion. In addition, because non-human animals showed high inter-individual variability in their sensitivity to the Solitaire array, inter-individual variability of human participants in the present experiment was analyzed as a further tool for comparative investigation.

## Materials and Methods

### Participants

Sixteen adult human volunteers (seven males, nine females) between the ages of 21 and 31 years (mean age 25.94 years) took part in the experiment. All had normal or corrected-to-normal vision. The task was approved by the ethics committee of the Department of General Psychology of University of Padova (Italy). All participants gave their informed consent prior to participating in the experiment, in accordance with the Declaration of Helsinki, and they received course credit for their participation.

### Apparatus

The testing setup included a personal computer, a keyboard, and a 17-in LCD color monitor. A height-adjustable chin rest was available to keep participants’ eyes at a constant comfortable distance from the monitor (60 cm). The task was conducted in a quiet and dimly illuminated room. In the relative numerosity task, participants were shown two arrays comprised of yellow and blue items. The two arrays were positioned on the left-center and right-center quadrants of the computer screen. In the numerosity identification task, a single array at a time was presented in the center of the screen.

To date, comparative studies on the Solitaire illusion used only two types of stimuli: dots (Agrillo et al., 2014a; Parrish et al., 2016; Miletto Petrazzini et al., under review) or food items (blue M&Ms and yellow cereals, Agrillo et al., 2014a). To assess whether this numerosity illusion may vary as a function of the type of visual array, both types of stimuli were shown: yellow and blue dots (dots were 0.4 cm in diameter), and pictures showing blue *M&M’s* and yellow cereal pieces taken from the study on chimpanzees (Agrillo et al., 2014a). Each set was included within a 7.5 cm × 7.5 cm green rectangle. In both tasks, they were not given any feedback throughout the experiment after the initial instructions.

### Relative Numerosity Task

Participants were given a two-option choice task from which they were required to select the array that they perceived to contain the larger number of blue items. Each array contained a combination of yellow and blue items. These items were either randomly arranged for control trials or arranged in a specific cross-pattern for the Solitaire trials. To prevent participants becoming biased toward responding to the overall number of items included in single arrays (blue + yellow items together), for each trial, the two arrays contained an equal number of yellow and blue items combined. But, these arrays were the inverse relation of one another with regard to specific quantities of each item type. For example, for a 13 vs. 16 control trial, each array would contain 29 items but one array would contain 13 blue items and 16 yellow items while the second array would contain 16 blue items and 13 yellow items. For this example, participants should select the array that contained more blue items even though the arrays had the same overall quantity. For test trials with Solitaire illusion, each array contained 16 blue and 16 yellow items, with one array presenting 16 blue items centrally located with 16 yellow items on the perimeter, and one array presenting 16 yellow items centrally located with 16 blue items on the perimeter.

**FIGURE 2:**
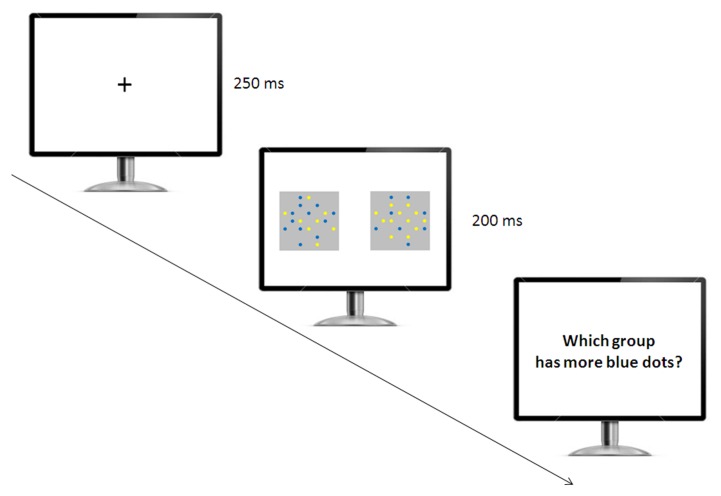
**Experimental setup used in the relative numerosity task.** The participants initially saw a fixation cross, and then two arrays of yellow and blue items appeared simultaneously. The participants were required to indicate which array had the larger number of blue items.

Participants were singly tested in the experimental room (**Figure 2**). They were instructed to select the array with the larger number of blue dots by pressing one of two color-coded buttons of the keyboard. A fixation cross appeared in the center of the screen for 250 ms, then two arrays of blue and yellow items were presented for a short amount of time (200 ms), a typical presentation time adopted to prevent verbal counting in non-symbolic numerical tasks with humans (Halberda et al., 2008; Agrillo and Piffer, 2012; Price et al., 2012; Agrillo et al., 2013, 2015b). We recorded accuracy in terms of selecting the array with the larger number of blue items for control trials, as well as the specific arrays selected on all trials. Participants were required to press the space bar to start the next block of trials. We included seven trial types, including six control trials and one Solitaire illusion trial (test trial). For the control trials, we introduced six quantity comparisons: 4 vs. 8, 8 vs. 12, 8 vs. 16, 10 vs. 16, 12 vs. 16, and 13 vs. 16.

Testing consisted of one session per participant, with 112 trials per session divided into two blocks of 56 trials. In one block, participants were presented with yellow and blue dots as stimuli; in the other block, they were shown pictures of food items that were presented in the study with chimpanzees (Agrillo et al., 2014a). The order of presentation of the two blocks was semi-randomized across participants. Each session included 16 trials of each trial type (the Solitaire trial type and the six control trial types). Arrays were extracted from a pool of four different alternative arrays for each trial type. However, because each array could be rotated on its side, there were four different orientations, for a total of 16 different pattern configurations (4 arrays × 4 rotations). Order of trial type was randomized, and the digital array (left or right) that contained the larger number of blue items was also randomized within session. For Solitaire test trials, we included eight trials in which the blue items were centrally located in the array on the left side and eight trials in which the blue items were located on the perimeter in the array on the left side.

Statistical analyses were carried out using SPSS version 23.0, and in some cases we applied the Holm–Bonferroni Correction Calculator (Gaetano, 2013).

### Numerosity Identification Task

Following completion of the relative numerosity task, the same participants began this task. Participants were presented with single arrays of inter-mixed yellow and blue items and were instructed to verbally report the number of blue items in each digital array for each trial as accurately as possible.

**FIGURE 3:**
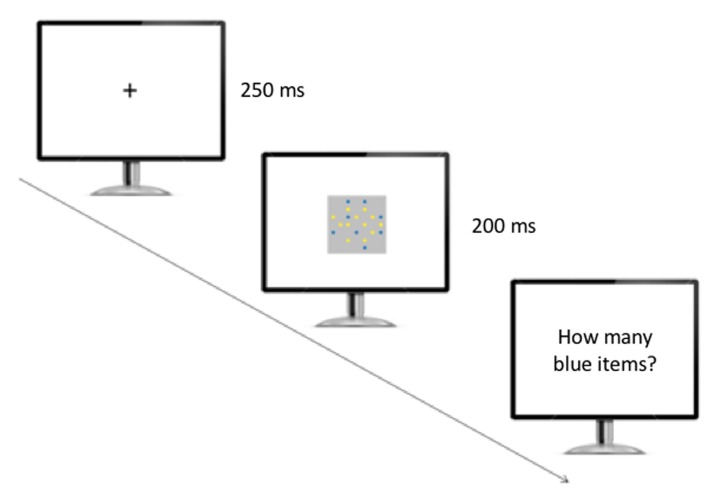
**Experimental setup used in the numerosity identification task.** The participants initially saw a fixation cross, and then a single array of yellow and blue items appeared on the screen. The participants were required to verbally report the number of blue items they believed they had seen.

After the presentation of a fixation cross (250 ms), a single array including blue and yellow items was presented for 200 ms, and participants were required to report aloud the number of blue items they thought they had seen (**Figure 3**). A microphone positioned in front of the participants recorded their response. Participants pressed the space bar to proceed to the next trial. No feedback was provided throughout the experiment. The session was broken into two equal blocks (48 trials per block), each of which was initiated when participants pressed the space bar to begin.

As in the previous task, two different types of stimuli were presented in the two blocks according to a semi-random sequence: yellow and blue dots, and pictures showing blue M&M’s and yellow cereal pieces taken from Agrillo et al. (2014a).

Participants’ estimation of 4, 8, 10, 12, and 13 blue items were classified as control trials, while estimations of 16 items arranged within the Solitaire pattern were considered as test trials. The number of yellow items in each array ranged from 8 to 16. Each control trial appeared 16 times; test trials also appeared 16 times (eight trials with blue items centrally located; eight trials with blue items located on the perimeter). Blocks of six trials were presented according to a semi-random sequence. We calculated the numerical distance (absolute value) between the presented number of items and the participants’ response as the dependent measure.

## Results

### Relative Numerosity Task

There was no difference in accuracy of selecting the array with the larger number of blue items as a function of the stimulus type (dots or pictures of food items) for control trials [dots: 0.92 ± 0.05, food item pictures: 0.91 ± 0.03, paired *t*-test *t*(15) = 0.94, *P* = 0.362] and Solitaire illusion test trials in terms of selection of the array that had the centrally located blue items [dots: 0.83 ± 0.12, food item pictures: 0.81 ± 0.12, *t*(15) = 1.06, *P* = 0.307]. As a consequence, in the following analyses we collapsed performance across the two types of stimuli.

#### Control Trials

**FIGURE 4:**
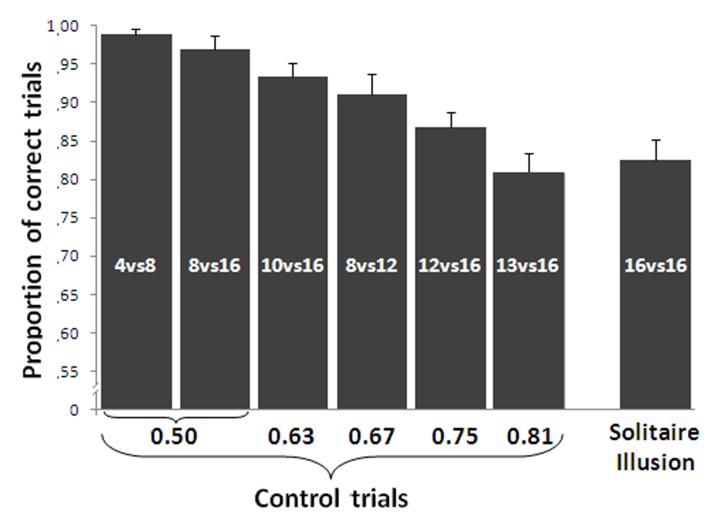
**Results of the relative numerosity task.** Accuracy in the control trials was significantly affected by the numerical ratio. In the Solitaire illusion trials, participants selected the array with the centrally located blue items significantly more often than the array with the blue items located on the perimeter. In the Solitaire illusion test, the *Y*-axis indicates the proportion of trials in which participants selected the array with centrally located blue items. Bars represent the standard error of the mean.

A repeated measures ANOVA with ratio (small set divided by large set) as a factor showed a main effect of ratio on the control trials such that accuracy decreased as ratio increased [linear contrast, *F*(1,15) = 71.442, *p* < 0.001, **Figure 4**]. Despite this predicted effect, a one sample *t*-test showed a significant discrimination at above chance levels for all numerical contrasts [4 vs. 8: *t*(15) = 57.46, *P* < 0.001; 8 vs. 16: *t*(15) = 24.78, *P* < 0.001; 8 vs. 12: *t*(15) = 15.70, *P* < 0.001; 10 vs. 16: *t*(15) = 24.73, *P* < 0.001; 12 vs. 16: *t*(15) = 18.64, *P* < 0.001; 13 vs. 16: *t*(15) = 11.94, *P* < 0.001].

#### Solitaire Illusion Test

Group analysis showed that participants significantly selected as more numerous the pattern where blue items were centrally located vs. when they were located on the perimeter [*t*(15) = 11.54, *P* < 0.001, Cohen’s *d* = 5.96, **Figure 4**]. This indicates that, as with past research (Frith and Frith, 1972; Agrillo et al., 2014a), adult humans in this experiment saw the array with blue items centrally located as being more numerous than the array with blue items at the perimeter.

To assess whether inter-individual differences existed in the perception of the illusion, we also performed individual analyses (see **Table 1**). Binomial tests with Holm-Bonferroni correction on the frequency of choices for the array in which blue items were centrally located showed that eight participants out of 16 (50%) showed a robust misperception of numerosity at the individual level (*P* < 0.05). Three participants showed a marginally significant choice in the same direction while the remaining five participants did not select one array more than chance.

**Table 1:** Individual performance of participants in test trials with Solitaire illusion of the relative numerosity task (corrected alpha level = 0.006).

Subject	Freq. choices for arrays with centrally located blue items	Total trials	Binomial test (Holm–Bonferroni correction)
1	15	16	*p* = 0.001^∗^
2	12	16	*p* = 0.077
3	12	16	*p* = 0.077
4	09	16	*p* = 0.804
5	13	16	*p* = 0.021
6	11	16	*p* = 0.210
7	15	16	*p* = 0.001^∗^
8	15	16	*p* = 0.001^∗^
9	13	16	*p* = 0.021
10	14	16	*p* = 0.004^∗^
11	15	16	*p* = 0.001^∗^
12	14	16	*p* = 0.004^∗^
13	14	16	*p* = 0.004^∗^
14	13	16	*p* = 0.021
15	15	16	*p* = 0.001^∗^
16	11	16	*p* = 0.210

*Asterisks denote a significant departure from chance*.

We further assessed how strongly the illusory effect occurred by comparing the response bias on test trials to the performance on control trials where there was a true difference in the number of blue items. A significant difference was found between the performance in the Solitaire pattern and that reported for the 0.50 ratio [paired *t*-test *t*(15) = 5.42, *P* < 0.001], 0.63 [*t*(15) = 3.09, *P* = 0.007] and the 0.67 ratio [*t*(15) = 2.36, *P* = 0.032]. However, no difference was found between the performance with the Solitaire pattern and that reported with the other larger ratios (all *P* > 0.226). This would suggest that the Solitaire illusion produces a rather substantial overestimation of the number of blue items in one array relative to the other.

### Numerosity Identification Task

Consistent with the previous task, there was no difference in the average discrepancy of the participants’ numerical estimates and the true array quantities as a function of the stimulus type for control trials [dots: 0.53 ± 0.20, food item pictures: 0.54 ± 0.21, paired *t*-test t(15) = 0.99, *P* = 0.336] and the Solitaire illusion test [dots: 2.58 ± 1.33, food item pictures: 2.57 ± 1.42, *t*(15) = 1.33, *P* = 0.203]. As a consequence, in the following analyses we collapsed performance across the two types of stimuli.

#### Control Trials

**FIGURE 5:**
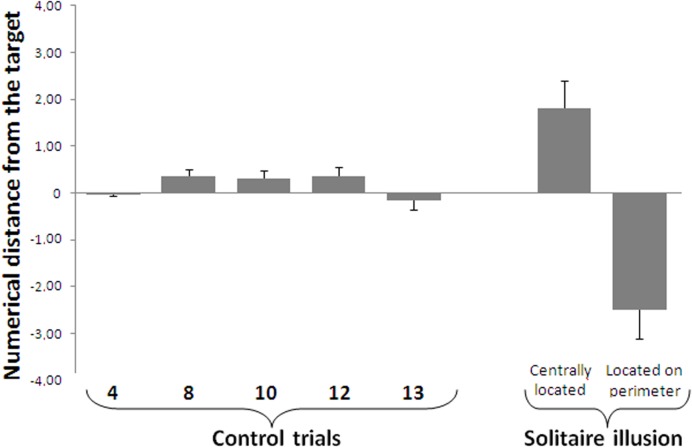
**Results of the numerosity identification task.** When required to name the exact number of blue items, participants’ accuracy decreased as a function of the true number of blue items. Negative values indicate an underestimation of the number of target items, while positive values reflect an overestimation of target items. For the Solitaire illusion trials, participants were less accurate when the blue items were located on the perimeter compared to when they were centrally located, although in both cases perceptual errors occurred. Bars represent the standard error of the mean.

A repeated measures ANOVA with true quantity of blue items in the array (4, 8, 10, 12, and 13) as a within-subjects factor showed a main effect of array size, meaning that accuracy decreased as the numerosity of the items to be estimated increased [linear contrast, *F*(1,15) = 71.44, *P* < 0.001, see **Figure 5**]. One sample *t*-tests comparing participants’ estimation with exact values of target numbers showed that participants were accurate in all control numbers except for estimating 8 [4: *t*(15) = 0.131, *p* = 0.897; 8: *t*(15) = 2.675, *p* = 0.017; 10: *t*(15) = 1.774, *p* = 0.096; 12: *t*(15) = 1.728, *p* = 0.105, 13: *t*(15) = 0.893, *p* = 0.386].

#### Solitaire Illusion Test

One sample *t*-tests showed that participants misperceived the numerosity of blue items both when they were centrally located [*t*(15) = 3.018, *p* = 0.007, *d* = 1.56] and when they were located on the perimeter [*t*(15) = 4.040, *p* = 0.001, *d* = 2.09]. These results indicate that the Solitaire arrangement disrupts quantity estimation for both Solitaire arrays, including overestimate of centrally clustered items and underestimation of items dispersed on the perimeter that determine numerosity illusions in both sub-sets. A paired *t*-test showed a significant difference between participants’ estimation of 16 blue items centrally located (mean ± standard deviation, 17.81 ± 2.33) and their estimation of 16 blue items located in the perimeter [13.51 ± 2.47, *t*(15) = 12.471, *p* < 0.0001, **Figure 5**].

To assess inter-individual differences in the perception of the illusion, we also performed individual analyses. In particular, we analyzed whether the estimation of each participant in the presence of illusory arrays was significantly different from test value (16). Thirteen participants perceived the illusion. However, seven of 16 participants (44%) misperceived the numerosity of blue items when they were centrally located, while 11 out of 16 (69%) misperceived the numerosity of blue items when they were in peripheral locations, suggesting that the illusory effect in numerical estimation is greater for items located at the perimeter. **Table 2** illustrates individual performance of each numerical target in all trial types.

**Table 2:** Individual performance of participants (numerical distance between participants’ estimation and correct value) in the numerosity identification task.

Subject	Control trials					Test trials			
	4	8	10	12	13	16 centrally located	*t*-test (Holm–Bonferroni correction)	16 located in the perimeter	*t*-test (Holm–Bonferroni correction)
**1**	0	-0.188	-0.500	0.813	0.188	4.500	*t*(7) = 7.937, *p* < 0.0001^∗^	-1.250	*t*(7) = -2.118, *p* = 0.072
**2**	-0.060	0.125	-0.750	-0.625	0.438	-0.870	*t*(7) = -1.369, *p* = 0.213	-4.250	*t*(7) = -11.613, *p* < 0.0001^∗^
**3**	-0.188	0.563	0.313	1	-1	-1.375	*t*(7) = -1.353, *p* = 0.218	-3.875	*t*(7) = -8.082, *p* < 0.0001 ^∗^
**4**	0	0.500	0.875	0.688	0.813	8.500	*t*(7) = 17.000, *p* < 0.0001^∗^	6.000	*t*(7) = 11.225, *p* < 0.0001^∗^
**5**	0.438	1	1.438	0.813	0.438	0.563	*t*(7) = 0.629, *p* = 0.549	-2.313	*t*(7) = -2.569, *p* = 0.037
**6**	0.250	0.813	0.563	1.250	0.500	0.250	*t*(7) = 0.552, *p* = 0.598	-2.750	*t*(7) = -2.814, *p* = 0.026
**7**	0.375	0.313	-0.188	1.750	0.438	1.188	*t*(7) = 2.049, *p* = 0.080	-2.938	*t*(7) = -2.898, *p* = 0.023
**8**	0	0.438	0.500	0	-0.875	2.125	*t*(7) = 9.379, *p* < 0.0001^∗^	-4.250	*t*(7) = -13.561, *p* < 0.0001^∗^
**9**	0	1	2	1	-1	3	*t*(7) = 7.099, *p* < 0.0001^∗^	-3.063	*t*(7) = -4.889, *p* = 0.002^∗^
**10**	0	0.500	-0.250	0.875	-0.688	2	*t*(7) = 4.000, *p* = 0.005^∗^	-4.250	*t*(7) = -6.561, *p* < 0.0001^∗^
**11**	0	0	0	0.438	0.813	0.250	*t*(7) = 0.266, *p* = 0.798	-4.375	*t*(7) = -7.744, *p* < 0.0001^∗^
**12**	-0.188	1	-0.250	0.188	-0.938	2.125	*t*(7) = 3.067, *p* = 0.018	-3.000	*t*(7) = -4.786, *p* = 0.002^∗^
**13**	-0.563	-0.313	0.063	-0.625	-1	3.000	*t*(7) = 3.642, *p* = 0.008	-2.063	*t*(7) = -4.123, *p* = 0.004 ^∗^
**14**	-0.188	-0.875	0.188	-0.625	-1	0.250	*t*(7) = 0.352, *p* = 0.732	-3.063	*t*(7) = -4.889, *p* = 0.002^∗^
**15**	0	1	0.500	-0.938	0.438	1.813	*t*(7) = 4.710, *p* = 0.002^∗^	-3.063	*t*(7) = -4.889, *p* = 0.002^∗^
**16**	0	0	0.500	-0.438	-0.188	1.688	*t*(7) = 4.333, *p* = 0.003^∗^	-1.375	*t*(7) = -3.274, *p* = 0.014

*Asterisks denote a significant departure from chance (test value = 16) in the presence of the illusory arrays (corrected alpha level = 0.006).*

The mean estimate when blue items were centrally located was 17.81, and the mean estimate when blue items were in the perimeter was 13.51. These two values allowed us to calculate a measure of the average relative difference in how the two arrangements were perceived by participants by calculating the ratio between the two perceived numerosities (13.51/17.81). The result indicated that items located on the perimeter were perceived to be only 76% as numerous as items that were centrally located.

**FIGURE 6:**
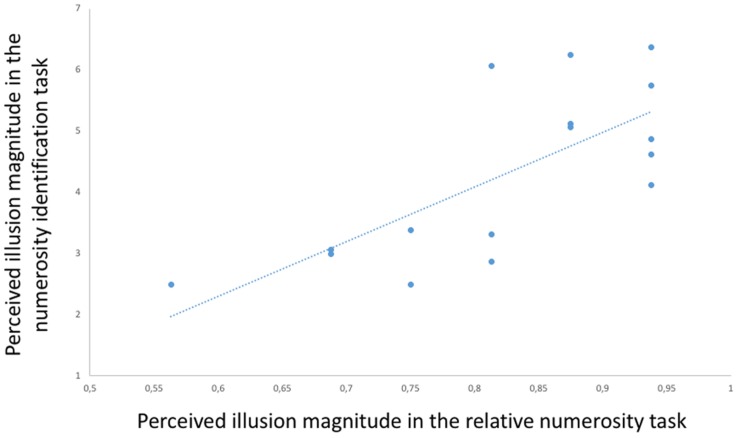
**Participants’ performance in the presence of the Solitaire illusion in the two tasks.** A positive correlation [*r*(15) = 0.726, *p* = 0.001] was found between the performance in the relative numerosity task (proportion of choices of centrally located blue items as more numerous than peripheral blue items) and the performance in the numerosity identification task (numerical distance in estimation of blue items centrally presented vs. peripherally presented).

Finally, to assess the consistency of Solitaire illusion perception in the two tasks, we correlated within the participants the performance in the presence of the illusory arrays in the relative numerosity task (proportion of choices for the array with blue items centrally located) with the performance in the numerosity identification task (numerical distance in estimation of blue items centrally located vs. located in the perimeter). This Pearson test showed a significant positive correlation [*r*(15) = 0.726, *p* = 0.001, **Figure 6**], indicating that relative susceptibility to the illusion in one task predicted relative susceptibility to the illusion in the other task.

## Discussion

This study had two aims: first, to test the existence of the Solitaire illusion (32 item version) in adult humans presented with the same experimental material used in comparative research (two arrays presented simultaneously made by dots and images of food items). Second, we assessed the strength of the Solitaire illusion in order to understand whether the subjective ratio generated by the illusion in adult humans was a ratio that could potentially be perceived in non-human species in terms of their quantitative acuity for discriminating sets. This information could help us to better interpret existing and future data in this area of research.

Consistent with the initial report of the Solitaire illusion among human adults and 8-year old children (Frith and Frith, 1972), adult participants in the current study misperceived the relative number of two identical types of items that differed only in color when presented in intermingled sets. Sets that contained items that were centrally located or clustered in the middle were overestimated relative to a set with an identical number of items that were instead located on the perimeter in smaller clusters. These results also are consistent with the pattern of performance reported for pre-school children (Parrish et al., 2016). We also have assessed this numerosity illusion among non-human primates, demonstrating more inconsistencies in performance patterns with chimpanzees failing to perceive the illusion and variable results among rhesus macaques and capuchin monkeys, with some individual monkeys showing the illusion in a human-like direction and others failing to do so (Agrillo et al., 2014a; Parrish et al., 2016). In attempts to shed light on the discrepancy in findings among humans and animals, we assessed the exact misperception of numerosity generated by the Solitaire arrangement among human adults, and the possibility that the numerical effects generated by this numerosity illusion are too subtle to be consistently detected by non-human animals.

In the relative numerosity task, we presented participants with a computerized two-choice forced discrimination task in which they were instructed to choose the set containing a larger number of blue items intermixed with identically sized yellow items. As expected, participants performed very well in control trials, choosing the array with more blue items when there was a true difference in the number of blue items across sets. The results show that performance was impacted as it should have been by the relative numbers of blue items in both choice arrays. As that difference increased, the discrimination became easier and the participants thus produced the expected ratio effect (Dehaene et al., 1993; Revkin et al., 2008). For Solitaire illusion trials, participants selected the array with the centrally located blue items at significantly higher rates than the other array with blue items located on the perimeter. Additionally, performance for the illusion probe trials was similar to that observed in control trials with numerical ratios larger than 0.67. These findings contribute to the existing literature on the Solitaire illusion to quantify the magnitude of this numerosity illusion, suggesting that the illusory array produces a substantial overestimation of the number of blue items in one array relative to the other.

Analyses on individual performance confirmed that the Solitaire illusion emerges robustly for some participants, but this illusion is not ubiquitous across human adults. As shown here, half of the participants (8 out of 16) showed clear evidence of the numerosity illusion with three additional participants exhibiting a marginally significant misperception. This result does not match with those reported in non-human animals, in which overall perception is sometimes reported at the group level but only weakly at the individual level (capuchin monkeys: Parrish et al., 2016; guppies: Miletto Petrazzini et al., under review). Hence, these data confirm that, although some individuals among some non-human animal species perceive the Solitaire illusion in the predicted direction, the strength of the illusion is likely to be stronger in our species with less inter-individual variability.

The brief presentation time of the arrays was necessary to avoid verbal counting and permit a comparison with the comparative literature in which counting is not a mechanism used by animals. However, one may argue that, given the complexity of the visual pattern presented and the limited presentation time of the arrays, some participants may not have compared the number of blue items in the two paired arrays, but rather might have focused on the number of blue and yellow items inside a single array. Participants were clearly instructed to focus on blue items only. However, even assuming that some participants may have adopted the alternative strategy, the pattern of data was not expected to change as the paired arrays contained the same number of yellow and blue items combined but in an inverse relation to one another. For example, in the 13 vs. 16 item discrimination, participants focusing on the internal ratio between blue and yellow items would have had to make the same discrimination in terms of numerical ratio (13 blue vs. 16 yellow items). The same could have happened with the illusory array (16 blue vs. 16 yellow items). Thus, we do not believe this alternative strategy might have affected our data.

In the numerosity identification task, we further assessed the nature by which human adults misperceive the Solitaire arrays by presenting participants with a single array containing intermixed yellow and blue items, with the task of estimating and reporting the exact number of blue items perceived on each trial. Participants accurately estimated target number in control trials. However, as expected, their accuracy decreased as the number of items to be estimated increased. Regarding the estimates of the illusion probe trials, participants misperceived the number of blue items when they were centrally located and when they were located in smaller clusters on the perimeter of the array. But, interestingly, group and individual analyses showed that participants were less accurate for reporting the number of blue items on the perimeter than for the centrally located blue items, suggesting that these items on the perimeter may be less well attended or apprehended secondarily to the central items as a function of their dispersed location. Follow-up research using eye-tracking data would be useful in demonstrating the validity of this hypothesis. This experiment also allowed for an estimate of the degree of over-estimation and under-estimation of items within the illusory array, with perimeter items perceived to be only 76% as numerous as items that were centrally clustered. Future studies are necessary to assess whether a similar ratio can be found with the other Solitaire arrays made by Frith and Frith (1972). Similarly, it would be interesting to extend this investigation to the regular-random numerosity illusion, which also emerges due to the relative spatial arrangement of stimuli within an array (Ginsburg, 1980).

One potential limit of the numerosity identification task is that the procedure is not commonly used with non-human animals. Indeed, most of the comparative literature investigated numerical abilities of vertebrates by using relative numerosity judgments (e.g., which array has more? Kilian et al., 2005; Beran, 2006; Miletto Petrazzini et al., 2015) or, to some extent, absolute numerosity categorizations (e.g., which array has three items? Nieder et al., 2002; Ditz and Nieder, 2015). In the present study, human participants were given a numerosity identification task, a type of procedure that has only rarely been used with animals previously trained to use symbolic numbers (Pepperberg, 1987, 1994; Boysen and Berntson, 1989; Biro and Matsuzawa, 2001). We cannot exclude that the subjective ratio generated by the illusion might be partially different by using other procedures more commonly adopted in comparative research.

Another potential limit of this task involves the lack of a condition in which participants had to estimate 16 dots not arranged in the Solitaire pattern. In the absence of this condition, we cannot exclude the possibility that the performance observed in the test trials (either 16 items centrally located or located in the perimeter) could be the result of the lower performance of participants in the presence of the highest numerosity presented in the whole task (16). We believe this is an unlikely circumstance, as the performance of centrally located items is completely opposite to that reported with items located in the perimeter, but future investigation should directly test this possibility.

When we analyzed the individual performance between the two tasks in the presence of the illusory arrays, we found a positive correlation, meaning that there was a general consistency among participants in Solitaire illusion perception with two different experimental paradigms. However, we also found some interesting variability. Four participants that did not show evidence of the illusion in the relative numerosity task showed a misperception of blue items in the numerosity identification task. Thus, the different pattern of data reported in these participants between the two experiments is intriguing. The relative numerosity task was characterized by the simultaneous presentation of two complex arrays made by yellow and blue dots for a short amount of time, a condition that might have engaged attentional resources and working memory differently compared to the numerosity identification task. It is possible that the type of task may have affected the sensitivity to illusory patterns. Given that different results sometimes have been reported among species that underwent different types of procedures for assessing illusions (e.g., Zollner illusion: Watanabe et al., 2011; Agrillo et al., 2014b; Ebbinghaus illusion: Parron and Fagot, 2007; Nakamura et al., 2008; Rosa Salva et al., 2013; Delboeuf illusion: Parrish et al., 2015), this hypothesis should be tested.

## Conclusion

In the current study we provided evidence of inter-individual variability in the perception of Solitaire illusion and provided a measure of the strength of this illusion in humans. Our data, however, are likely to have substantial implications also for comparative research. The fact that the illusory ratio seems to be roughly equal to 0.76 could help us to better understand the existing literature and future studies with non-human animals. As chimpanzees (e.g., Beran, 2004), rhesus monkey (e.g., Beran, 2007), capuchin monkeys (e.g., Evans et al., 2009) and guppies (Bisazza et al., 2014; Miletto Petrazzini et al., 2015) are able to discriminate 0.76 ratio in different experimental contexts, the performance differences reported between humans and these species may be more related to true differences in perceptual mechanisms (e.g., difference in grouping mechanisms) or individual differences in discriminatory thresholds affecting numerical estimation, instead of being the result of lower numerical acuity of non-human animals. Future studies on other species taking into account the illusory ratio here reported are welcome to help us understand why the Solitaire illusion appears to be more elusive in animal species than other numerical illusory phenomena described in the comparative literature.

## Author Contributions

All authors listed have made substantial, direct and intellectual contribution to the work, and approved it for publication.

## Conflict of Interest Statement

The authors declare that the research was conducted in the absence of any commercial or financial relationships that could be construed as a potential conflict of interest.
